# Long-Term Protection Elicited by a DNA Vaccine Candidate Expressing the prM-E Antigen of Dengue Virus Serotype 3 in Mice

**DOI:** 10.3389/fcimb.2020.00087

**Published:** 2020-03-17

**Authors:** Kaihao Feng, Xiaoyan Zheng, Ran Wang, Na Gao, Dongying Fan, Ziyang Sheng, Hongning Zhou, Hui Chen, Jing An

**Affiliations:** ^1^Department of Microbiology and Parasitology, School of Basic Medical Sciences, Capital Medical University, Beijing, China; ^2^Beijing Tropical Medicine Research Institute, Beijing Friendship Hospital, Capital Medical University, Beijing, China; ^3^Yunnan Provincial Key Laboratory of Vector-borne Disease Control and Research, Yunnan Institute of Parasitic Diseases, Pu'er, China; ^4^Center of Epilepsy, Beijing Institute for Brain Disorders, Beijing, China

**Keywords:** dengue virus, DNA vaccine, prM-E, immunization, cross-protection

## Abstract

Dengue virus (DENV) is the causative agent of dengue, and its incidence has increased 30-fold in the past five decades. Among the four cocirculating serotypes, DENV3 is associated with an increased number of severe infections and has become widespread. Vaccination is the mainstay of prevention in reducing disease burden. Previously, the protective efficacy of DNA vaccine candidates toward DENV1, 2, and 4 was confirmed in mice. In this study, a DNA vaccine candidate (pVAX1-D3ME) expressing the prM and E proteins of DENV3 was constructed, and then the immunogenicity and protection were assessed in mice to further develop a tetravalent dengue vaccine. Moreover, the cross-reactive immune responses against the other three serotypes were investigated. The results showed that three doses of 50 μg of pVAX1-D3ME were sufficient to induce strong antigen-specific T cell responses and robust and consistent neutralizing antibodies. Additionally, immunization with pVAX1-D3ME offered protective immunity against not only DENV3 but also the other three serotypes, which could be observed even after 12 months. This study shows great promise for the further evaluation of a dengue tetravalent DNA vaccine candidate in large animal models, including non-human primates.

## Introduction

Dengue virus (DENV) is the cause of dengue, which is widespread in tropical and subtropical countries and affects 390 million people annually (Kularatne, 2015). The incidence of dengue infection has increased rapidly over the last 50 years along with the geographic expansion of the disease (Kraemer et al., 2019). It is estimated that Asia bore 70% of the disease burden (Bhatt et al., 2013).

DENV belongs to the genus *Flavivirus* of the family *Flaviviridae*. Serologically, DENV can be subclassed into four distinct but closely related serotypes: DENV1, DENV2, DENV3, and DENV4. Infection with any one serotype presents with similar and indistinguishable clinical manifestations that range from asymptomatic, undifferentiated fever to dengue fever, and more severe life-threatening diseases, including dengue hemorrhagic fever and dengue shock syndrome.

It should be noted that DENV3 from Southeast Asia area causes the greatest percentage of severe cases in primary infection (Soo et al., 2016) and that DENV3 genotype III has been associated with a widespread global distribution of dengue fever (Tan et al., 2018). During 2016, a change in the circulating serotype occurred, leading to dominance of DENV3 in India (Parveen et al., 2019). In 2017–2018, in Dhaka, Bangladesh, the largest dengue outbreak with a high frequency of severe dengue cases and high fatality could be traced back to the reemergence of DENV3 (Shirin et al., 2019).

In China, DENV3 was the first serotype documented in Guangdong in 1978 and was later isolated in Zhejiang in 2009 and in Yunnan in 2013, including from severe cases (Lai et al., 2015). In 2013, the first dengue outbreak in central China was reported in Henan Province in the northern temperate regions and was characterized by DENV3 predominance. The northward shift of the dengue epidemic region is a warning that the endemic range has expanded geographically in China (Huang et al., 2014; Lai et al., 2015). In addition, reemerging DENV3 necessitates immediate public health attention.

Currently, there is no widely used effective, specific prevention, and therapy against this severe disease. To resolve this significant, international public health problem, considerable effort has been directed toward the development of safe dengue vaccines. A number of candidates have been reported, including inactivated vaccines, live-attenuated vaccines, DNA vaccines, and subunit protein vaccines (Eckels and Putnak, 2003; Whitehead, 2016; Bustos-Arriaga et al., 2018; Manoff et al., 2019; Prompetchara et al., 2019; Wang et al., 2019). Of great concern, the first and only licensed dengue vaccine, Dengvaxia, had been approved for use in endemic areas owing to its higher efficacy among participants vaccinated at age ≥9 years (Ferguson et al., 2016; Henein et al., 2017); however, a *post-hoc* analysis of safety and efficacy reported a higher risk of severe dengue attack and hospitalization in vaccinated persons who had not been exposed to dengue (Sridhar et al., 2018). There continues to be a strong and urgent public health need for effective preventive interventions against dengue.

DENV has a single-stranded, positive-sense RNA genome containing a single open reading frame that encodes three structural (capsid, C; premembrane, prM; and envelope, E) and seven non-structural (NS1, NS2A, NS2B, NS3, NS4A, NS4B, and NS5) proteins. Exposed on the surface of mature DENV particles, the E glycoprotein protein is rich in immunological epitopes and contributes to the generation of effective protective immunity (Putnak et al., 2003; Lin et al., 2012). The E protein assembly is dependent on the prM protein expression (Oliveira et al., 2017). Therefore, both the prM and E proteins have become major antigen targets for vaccine design and development against DENVs and other flaviviruses (Guirakhoo et al., 2001; Mellado-Sanchez et al., 2005; Osorio et al., 2014).

In our previous work, DNA vaccine candidates encoding the prM and E proteins of DENV1 (Zheng et al., 2017), DENV2 (Chen et al., 2016), or DENV4 (Sheng et al., 2019) could protect mice from lethal corresponding virus challenge. In this study, another DNA vaccine, pVAX1-D3ME expressing the prM and E proteins of DENV3, was constructed in the same vector pVAX1, which is the unique America FDA-approved vector in clinical trial. The breadth of the humoral and cellular immune responses elicited by the vaccine candidate was investigated. Moreover, its short-term and long-term protective efficacies against DENV3 as well as the other three DENV serotypes were evaluated in a mouse model. The results indicated that three doses of pVAX1-D3ME via *in vivo* electroporation induced effective humoral and cellular immune responses and significantly protected mice against lethal DENV3 challenge. Moreover, immunization with pVAX1-D3ME also provided cross-reactive protection against DENV1, DENV2, or DENV4 challenge. This work will be of particular importance for the ongoing effort to develop a tetravalent dengue vaccine.

## Materials and Methods

### Animals and Ethics Statement

Adult BALB/c mice were purchased from Vital River Laboratory Animal Technology Co., Ltd. (Beijing, China). One-day-old BALB/c mice used for the neutralization assay were produced by adult female mice. The animals were maintained in specific-pathogen-free environments. The protocol for animal experiments was approved by the Institutional Animal Care and Use Committee of Chinese Capital Medical University (ethics approval number AEEI-2015-066). *In vitro* and *in vivo* infections were performed in the biosafety level 2 laboratory. All animal experiments were performed under diethyl ether anesthesia. All efforts were made to minimize suffering.

### Cells and Viruses

Vero cells (ATCC CRL-1586) were cultured in minimum essential medium with 5% fetal bovine serum and 1% penicillin–streptomycin solution at 37°C with 5% CO_2_. *Aedes albopictus* C6/36 cells (ATCC CRL-1660) were grown in RPMI 1640 medium supplemented with 10% fetal bovine serum at 28°C.

The Hawaii strain of DENV1, the H87 strain of DENV3, and the H241 strain of DENV4 were provided by the Guangdong Provincial CDC. The Tr1751 strain of DENV2 was isolated from a patient with dengue fever. Viruses were propagated in C6/36 cells and titered in Vero cells by plaque forming assay.

Purified DENV3 particles were harvested from DENV3-infected C6/36 cells and concentrated by 8% polyethylene glycol precipitation. Then, DENV3 particles were purified from clarified extracts by ultracentrifugation.

### Construction and Identification of pVAX1-D3ME

The *prM* and *E* regions of the H87 strain of DENV3 (GenBank accession number AB609590, nucleotides 365–2,413 bp) were amplified by PCR using the forward primer (5′-CGG ATC GCT AGC ATG GCG ATG CTG AGC ATT ATC AAC-3′) and reverse primer (5′-CAC ACA GGA TCC TTA AGC TTG CAC CAC GGC TCC CAG ATA-3′). Subsequently, the amplified fragment was subcloned into the pVAX1 vector (Invitrogen, USA) using *Nhe* I and *Bam*H I restriction sites, and the recombinant plasmid was named pVAX1-D3ME, which was confirmed by double-enzyme digestion and DNA sequencing. The pVAX1 vector served as the negative control.

### Indirect Immunofluorescence (IFA) Staining

pVAX1-D3ME or pVAX1 was transfected into Vero cells using Lipofectamine 2000 (Thermo Fisher Scientific, USA). Transfected cells were fixed with 4% paraformaldehyde, permeabilized with 0.2% Triton X-100 in PBS, and blocked with 1% bovine serum albumin. DENV3-infected mouse serum (1:100) and FITC-conjugated goat anti-mouse IgG (1:200, Immunotech, France) were used as the primary and secondary antibodies, respectively. The cells were examined and imaged under a fluorescence microscope (Olympus BX61, Japan).

### Western Blotting

pVAX1-D3ME or pVAX1 was transfected into Vero cells using Lipofectamine 2000 (Thermo Fisher Scientific, USA). Seventy-two hours after transfection, the supernatant and lysate were collected and fractionated by 12% SDS-PAGE and transferred to a polyvinylidene difluoride membrane according to the manufacturer's recommendation (Millipore, USA). The membrane was blocked with 5% bovine serum albumin for 2 h and incubated with anti-flavivirus E protein monoclonal antibody (1:50, D1-4G2-4-15 hybridoma supernatant, ATCC HB-112) at 4°C overnight, and then probed with goat anti-mouse HRP secondary antibody (1:4,000, abcam, China) for 1 h. Blots were developed with chemiluminescent HRP substrate peroxide solution mix (Millipore, USA) and visualized with Li-Cor Odyssey CLx imaging system.

### Immunization

**Figure 1:**
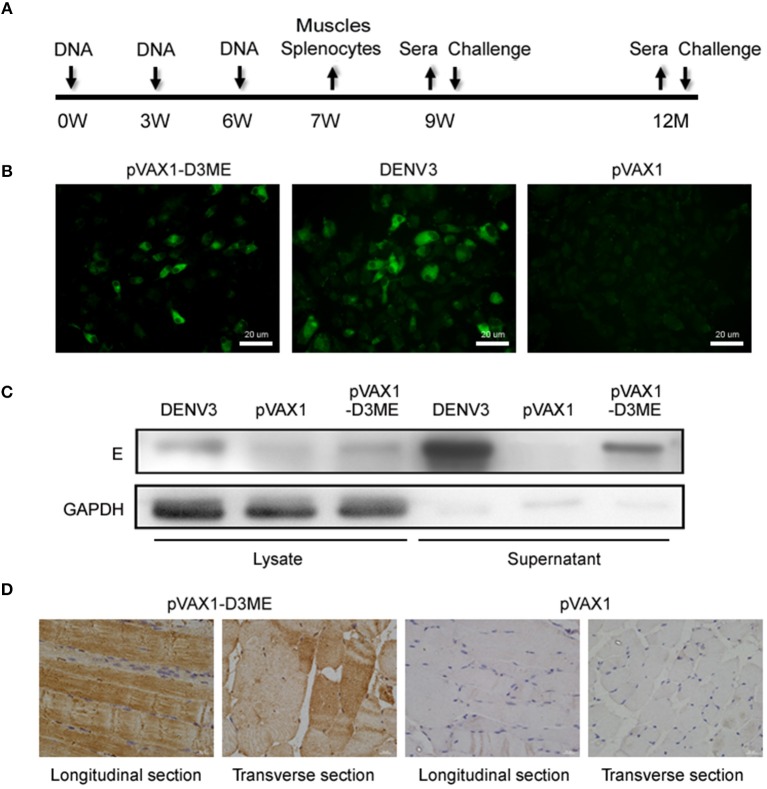
The immunization and sample collection schedule, and *in vitro* and *in vivo* expression of the prM-E proteins. **(A)** Diagram of immunization, sample collection and challenge in BALB/c mice. The mice were vaccinated at weeks 0, 3, and 6. Splenocytes and muscle tissues were obtained at the seventh week, and sera were collected at the ninth week, the sixth month, and the 12th month. The mice were challenged with DENVs at the ninth week and the 12th month. **(B)** *In vitro* expression of the prM-E proteins in plasmid-transfected Vero cells detected by IFA. DENV3-infected Vero cells served as the positive control. **(C)** The cell lysate and the supernatant of plasmid-transfected Vero cells were collected at 72 h after transfection and *in vitro* expression of the E protein was detected by Western blotting. DENV3-infected Vero cells served as the positive control. GAPDH served as the loading control. **(D)** *In vivo* expression of the prM-E protein at the injection site in muscle tissues detected by IHC.

Six-week-old female BALB/c mice were randomly separated into two groups. A total of 50 μg of pVAX1-D3ME or pVAX1 was injected into the quadriceps muscle, followed by six electric pulses (36 V, 10 ms) using a gene delivery device (Terasa Healthcare Sci-Tech, China). The mice were boosted twice at three-week intervals with the inoculation. The immunization and sample collection schedule are shown in Figure 1A.

### Immunohistochemistry (IHC) Staining

The thigh muscles were isolated one week after the final immunization. Paraffin sections were processed by conventional techniques as described previously (Chen et al., 2016). Convalescent serum derived from a DENV3-infected mouse was used at a 1:200 dilution as the primary antibody. HRP-conjugated goat anti-mouse IgG (Santa Cruz Biotechnology, USA) was diluted 1:2,000 and used as the secondary antibody.

### Plaque Reduction Neutralization Test (PRNT)

The neutralizing antibody (NAb) titer against DENV1–4 was determined by PRNT. Serum samples were heated at 56°C for 30 min to inactivate complement and then serially diluted from 1:10 to 1:1,280. Next, 100 μl of diluted sera was mixed with 100 μl of DENV suspension containing 50 plaque-forming units (PFU) and then incubated at 37°C for 1 h. The mixture was added to a confluent monolayer of Vero cells in a 24-well plate and incubated at 37°C for another hour under gentle rocking every 15 min. After washing, infected Vero cells were overlaid with medium containing 1.05% methylcellulose, followed by incubation at 37°C for 5–8 days. Finally, plaques were visualized by crystal violet diluent and counted. The reciprocal of the highest dilution that yielded a 50% reduction in the average number of plaques compared with the virus control wells was calculated as the neutralization titer (PRNT_50_).

### Enzyme-Linked Immunospot (ELISPOT) Assay

The cytokines IFN-γ and IL-4 secreted by splenocytes were captured by antibodies and visualized as spots based on a colorimetric reaction according to the manufacturer's (BD, USA) recommendations. In brief, 96-well-filtration plates (Millipore, USA) were coated with the capture antibodies at 4°C overnight and then blocked with 1% bovine serum albumin at 37°C for 2 h. Splenocytes isolated from immunized mice were aliquoted at 5 × 10^5^ cells/well and stimulated with purified DENV3 particles at a concentration of 5 μg/well at 37°C for 48 h. After incubation with a biotinylated detection antibody, the spots were visualized by adding streptavidin-HRP and AEC substrate and finally numerated automatically using an ELISPOT analyzer (CTL, USA). Splenocytes cocultured with concanavalin A served as a positive control, and those cultured with RPMI 1640 medium served as a negative control.

### Flow Cytometry Analysis

All antibodies for flow cytometry were purchased from BD Biosciences, USA. A total of 2 × 10^6^ splenic lymphocytes were blocked with rat anti-mouse CD16/CD32 monoclonal antibody for 30 min. For surface staining, all cells were stained with anti-CD3e-FITC, anti-CD8-APC-H7, anti-CD44-APC, and anti-CD62L-PE at a standard dilution. However, for intracellular cytokine staining, the splenocytes were stimulated with 1 μg of purified DENV3 particles for 24 h, and brefeldin A (10 μg/ml, Sigma, USA) was added at the end of the six-hour incubation. After staining with anti-CD3e-FITC, anti-CD8-APC-H7, and anti-CD11a-APC, the splenocytes were fixed and permeabilized using the BD Cytofix/Cytoperm kit and stained with anti-IFN-γ-PE. Finally, the stained cells were read on a DxFLEX flow cytometer (Beckman Coulter, USA) and analyzed by CytExpert software (version 1.2).

### *In vitro* Neutralizing and Passive Protective Effects of Immune Sera on Neonatal Mice

After heat inactivation, pooled sera isolated from the DNA-immunized mice were mixed with live DENV3 followed by incubation at 37°C for 1 h. Subsequently, 10 μl of the sera–virus mixture containing 100 PFU of DENV3 was gently injected intracranially into one-day-old neonatal mice. The body weight and survival rate were monitored daily for 14 days.

### Active Protection Against DENV1–4 in DNA-Immunized Mice

Mice were challenged intracerebrally with a lethal dose of DENV1, DENV2, DENV3, or DENV4. Pathological symptoms and body weight were monitored daily for 27 days. Pathological symptoms were recorded as the mean clinical sign scores: 0 = healthy; 1 = ruffled hair or hunchbacked appearance; 2 = asthenia, wasting, or bradykinesia; 3 = forelimb or hindlimb weakness; 4 = paralysis or moribundity; and 5 = death. Body weight changes were reported as percentages compared to those on day 0. The percentage was determined by 100% × (weight after challenge)/(weight before challenge). The survival rate was reported as the percentage of survivors.

### Statistical Analysis

Statistical analysis was conducted with SPSS 17.0 or GraphPad Prism 6 software. Geometric mean titers (GMTs) of NAbs were calculated as log-transformed reciprocal titers. Weight changes and clinical sign scores were analyzed by repeated measures analysis of variance. Kaplan–Meier survival curves were plotted and evaluated statistically by the log-rank test. The data of PRNT, ELISPOT, and flow cytometry analysis were compared using one-way analysis of variance. *p* < 0.05 were considered statistically significant: ^*^p < 0.05; ^**^p < 0.01; and ^***^p < 0.001.

## Results

### *In vitro* and *in vivo* Expression of the prM-E Protein

To test the *in vitro* expression of the recombinant plasmid in eukaryotic cells, Vero cells were transfected with pVAX1-D3ME or pVAX1, and the prM-E proteins were examined by IFA. As shown in Figure 1B, cells transfected with pVAX1-D3ME exhibited intense green fluorescence signals, and the transfection rate was more than 50%. In contrast, no specific fluorescence signal was observed in pVAX1-transfected cells. Furthermore, the expression of the recombinant plasmid was confirmed by Western blotting with the antibody 4G2, which recognizes the E antigen. Specific expression was detected not only in the lysate of pVAX1-D3ME-transfected cells but also in the supernatant (Figure 1C).

Moreover, to confirm the *in vivo* expression of the recombinant plasmid, mice were inoculated with plasmids three times at 3-week intervals, and the quadriceps femoris muscles were collected 1 week after the last immunization and tested by IHC. As shown in Figure 1D, in both the longitudinal and the transverse sections, the specific expression of the target protein was detected in the pVAX1-D3ME-inoculated muscle tissue, confirming that pVAX1-D3ME was effectively expressed *in vivo* and could be used in subsequent experiments.

### DENV3-Specific Humoral Immune Response

**Figure 2:**
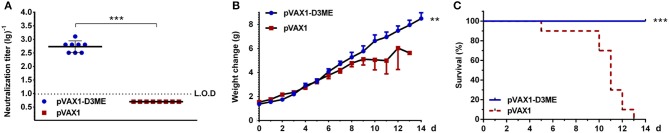
Humoral immune response against DENV3 in mouse sera. Sera were collected at the ninth week after the immunization. **(A)** Endpoint titers (*n* = 8) of DENV3-specific NAbs were detected by PRNT_50_ and recorded as GMT ± SD. The limit of detection (L.O.D.) depicted as a dotted line represents the lowest dilution that the experiment could detect. **(B,C)** *In vitro* neutralizing activity and passive protective effect of immunized sera on suckling mice (*n* = 13 in the pVAX1-D3ME group, *n* = 10 in the pVAX1 group). The pooled sera mixed with live DENV3 were transferred into 1-day-old suckling mice. The mice were monitored daily for 14 days. **(B)** Body weight change from day 0. **(C)** The survival rate is shown as the percentage of survivors. **p < 0.01; ***p < 0.001.

First, to evaluate the DENV3-specific antibody response triggered by pVAX1-D3ME, sera were collected from mice three weeks after the last immunization. The neutralization titers were assessed using *in vitro* PRNT. As expected, the sera of control mice failed to neutralize DENV3. In contrast, the PRNT_50_ GMT of pVAX1-D3ME-immunized sera was 1:538.17 (Figure 2A), suggesting a robust humoral immune response against DENV3.

Second, the protective efficacy of NAbs in the collected sera was evaluated in a neonatal mouse model. As shown in Figures 2B,C, the suckling mice that received sera from the pVAX1-D3ME group grew steadily with increasing body weights. The average body weight was 6.95 ± 0.42 g at day 11. In contrast, the control suckling mice showed a slow increase in body weight, and the average value was only 4.99 ± 1.11 g at day 11. By the end of the observation period, all of the suckling mice in the control group were dead, but 100% of the suckling mice in the pVAX1-D3ME group survived. Statistical analysis of the overall body weight and survival rate showed that there were significant differences between the two groups (^**^p < 0.01 and ^***^p < 0.001). Overall, pVAX1-D3ME-immunized sera provided suckling mice effective neutralization against DENV3 challenge and, to some extent, inhibited or reduced viral pathogenicity and delayed disease progression in suckling mice.

### DENV3-Specific Cellular Immune Response

**Figure 3:**
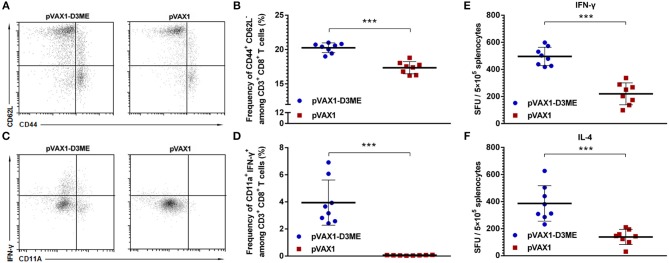
Cellular immune response against DENV3 in mouse splenocytes. Splenocytes were isolated 1 week after the final immunization. **(A–D)** The antigen-experienced CD8^+^ T cell response was assayed by flow cytometry (*n* = 8). **(A)** Representative images of CD44^+^ CD62L^−^ T cells (gated on CD3e^+^ CD8^+^ T cells). **(B)** Quantification of the frequency of CD44^+^ CD62L^−^ CD8^+^ T cells. The data are expressed as the mean percentage ± SD from three independent experiments. **(C)** Representative images of CD11a^+^ IFN-γ^+^ T cells (gated on CD3e^+^ CD8^+^ T cells). **(D)** Quantification of the frequency of CD11a^+^ IFN-γ^+^ CD8^+^ T cells. The data are expressed as the mean percentage ± SD from three independent experiments. **(E,F)** Splenocyte-secreted cytokines detected by ELISPOT assay (*n* = 8). The number of cytokine-positive cells is recorded as the mean spot-forming unit (SFU)/5 × 10^5^ splenocytes ± SD. ***p < 0.001.

As well-known, once T cells were activated, the expression of CD44 increased and that of CD62L decreased. CD44^+^ and CD62L^−^ were used to mark the effective memory T cells in this study. As shown in Figures 3A,B, after vaccination, the percentage of CD44^+^ CD62L^−^ cells within CD8^+^ T cells in the pVAX1-D3ME group was significantly higher than that in the pVAX1 group (^***^p < 0.001). This result indicated that the pVAX1-D3ME vaccination effectively improved the activation of effector memory T cells.

Similarly, the memory T cells generated after vaccination might play an essential role in long-term protection. Memory T cells can rapidly gain effector function to kill infected cells and/or secrete IFN-γ to inhibit the replication of viruses (Kaech et al., 2002; Zellweger et al., 2015). Murine CD11a^+^ CD8^+^ T cells are capable of mounting vigorous recall responses upon a secondary antigenic challenge (Rai et al., 2009). Therefore, combining the analysis with CD11a, a surface marker of antigen-experienced CD8^+^ T cells, we used flow cytometry to analyze changes in the percentage of memory CD8^+^ T cells expressing IFN-γ and CD11a in immunized mice. As shown in Figures 3C,D, after gating on CD3^+^ CD8^+^ T cells, an average 3.95 ± 1.56% proportion of CD11a^+^ IFN-γ^+^ cells was found in the pVAX1-D3ME group, which was ~60-fold higher than that in the pVAX1 group (^***^p < 0.001).

Taken together, these results indicated that pVAX1-D3ME induced a rapid and strong antigen-specific CD8^+^ T cell immune response.

### Cytokine Generation

One week after the last immunization, splenic lymphocytes were isolated and restimulated *in vitro* with the DENV3 antigen to detect the level of splenocyte-derived cytokines. As shown in Figures 3E,F, the levels of both IFN-γ and IL-4 were markedly higher in the pVAX1-D3ME group than those in the control group (^***^p < 0.001). The results suggested that pVAX1-D3ME vaccination was characterized by an elevated production of cytokines, further reflecting the activation of the immune system.

### Active Protection Against DENV3 Challenge

**Figure 4:**
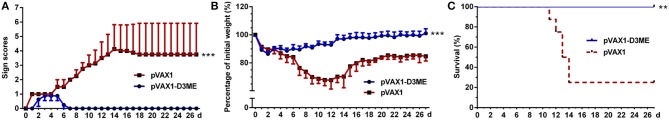
Short-term active protective immunity against DENV3 challenge (*n* = 8). Mice were challenged with DENV3 at the ninth week after the immunization and monitored daily for 27 days. **(A)** Pathological symptoms recorded as the mean clinical sign scores. **(B)** Percentage of body weight from day 0. **(C)** Survival rate shown as the percentage of survivors. Results are representative of three independent experiments. **p < 0.01; ***p < 0.001.

Three weeks after the final vaccination, mice were challenged with a lethal dose of DENV3. As shown in Figure 4, obvious illness signs appeared on the sixth day after challenge in the control group treated with pVAX1 (Figure 4A), and the clinical scores progressively increased. Moreover, body weight continuously decreased, and weight loss was >30% within 12 days (Figure 4B). Finally, 75% (6/8) of the control mice died (Figure 4C). In contrast, compared with the control mice, the pVAX1-D3ME-immunized mice showed transient and <15% body weight loss (^***^p < 0.001), with imperceptible illness symptoms (^***^p < 0.001). Moreover, all of the mice (8/8) survived from the lethal viral challenge (^**^p < 0.01), suggesting that three doses of pVAX1-D3ME were sufficient to provide full protective immunity against DENV3 infection.

### Long-Term DENV3-Specific NAb Response and Active Protection Against DENV3 Challenge

**Figure 5:**
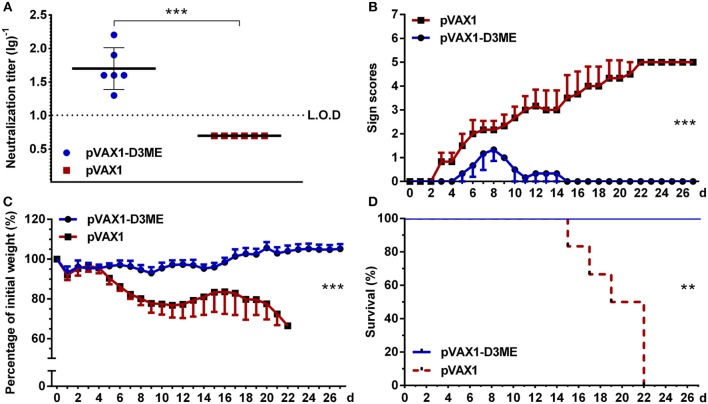
Long-term NAb response and active protective immunity against DENV3 challenge at the 12th month after the immunization (*n* = 6). **(A)** Endpoint titers of DENV3-specific NAbs in sera were detected by PRNT_50_ and recorded as GMT ± SD. The L.O.D. depicted as a dotted line represents the limit of detection of the assay. **(B–D)** Mice were challenged with DENV3 and monitored daily for 27 days. **(B)** Pathological symptoms recorded as the mean clinical sign scores. **(C)** Percentage of body weight from day 0. **(D)** Survival rate shown as the percentage of survivors. **p < 0.01; ***p < 0.001.

Moreover, at the 12th month after the immunization, the DENV3-specific NAb in pVAX1-D3ME-immunized sera were maintained at 1:50.4 (Figure 5A), suggesting a long-term humoral immune response against DENV3. When the mice were challenged with a lethal dose of DENV3, all of the pVAX1-D3ME-immunized mice (6/6) survived (^**^p < 0.01), with imperceptible symptoms (^***^p < 0.001), and body weight loss (^***^p < 0.001), as compared with the control mice (Figures 5B–D). The results suggested that three doses of pVAX1-D3ME triggered persistent NAb response and long-term effective protection against DENV3 infection.

### Cross-Reactive NAb Responses and Protection Against Other DENV Serotypes

**Figure 6:**
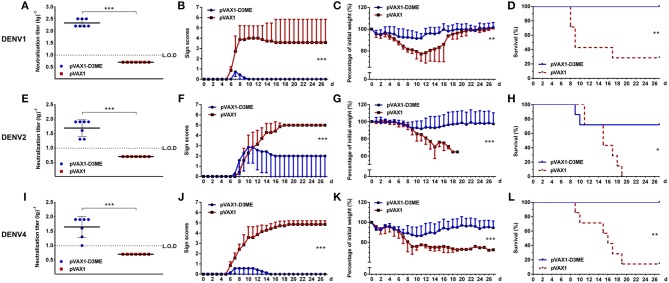
Short-term cross-reactive NAb response and protection against DENV1 **(A–D)**, DENV2 **(E–H)**, and DENV4 **(I–L)** at the ninth week after the immunization (*n* = 7). **(A,E,I)** Endpoint titers of cross-reactive NAbs in sera were detected by PRNT_50_ and recorded as GMT ± SD. The L.O.D. depicted as a dotted line represents the limit of detection of the assay. **(B–D,F–H,J–L)**. Mice were challenged with DENVs and monitored daily for 27 days. **(B,F,J)** Pathological symptoms recorded as the mean clinical sign scores. **(C,G,K)** Percentage of body weight from day 0. **(D,H,L)** Survival rate shown as the percentage of survivors. Results are representative of three independent experiments. *p < 0.05; **p < 0.01; ***p < 0.001.

The sera were collected three weeks after the last immunization to determine the short-term cross-reactive NAb responses. As shown in Figures 6A,E,I, the PRNT_50_ GMTs against DENV1, DENV2, and DENV4 were 1:215.34, 1:48.76, and 1:44.16, respectively, which showed cross-reactive neutralizing capability when compared with those of the corresponding controls (^***^p < 0.001).

Next, we investigated whether cross-reactive immunity could protect pVAX1-D3ME-immunized mice from challenge with other DENV serotypes. The kinetic changes in symptom scores, body weight, and survival rates are shown in Figure 6. Three weeks after the last immunization, after challenge with DENV1 and DENV4, 100% survival rates with relatively mild symptoms (Figures 6B,J, ^***^p < 0.001) as well as limited body weight loss (Figure 6C, ^**^p < 0.01 and Figure 6K, ^***^p < 0.001) were observed in the pVAX1-D3ME-immunized mice, whereas the survival rates were 28.6% (2/7, Figure 6D, ^**^p < 0.01) against DENV1 and 14.3% (1/7, Figure 6L, ^**^p < 0.01) against DENV4 in the control mice, which were accompanied with obvious illness signs and body weight loss.

Meanwhile, we observed that, when the mice were infected with DENV2, the pVAX1-D3ME-immunized group showed <10% body weight loss, slight illness signs, and a 71.4% survival rate (5/7). Although all of the abovementioned parameters were significantly improved compared to those in the pVAX1 group (25% body weight loss, severe illness signs, and 100% mortality) (Figures 6F,G, ^***^p < 0.001; Figure 6H, ^*^p < 0.05), the protective efficiency against DENV2 was not complete. These results indicated that the vaccine candidate pVAX1-D3ME could provide full protection against lethal DENV1 and DENV4 infection as well as partial protection against DENV2 infection.

### Long-Term Cross-Reactive NAb Responses and Protection Against Other DENV Serotypes

Furthermore, the persistence of the long-term cross-reactive NAb was investigated. At the sixth month after immunization, the respective cross-reactive NAb titers declined to 1:80, 1:26.92, and 1:32.81 (figure not shown) against DENV1, DENV2, and DENV4, which still showed cross-reactive neutralizing capability compared with those of the corresponding controls (^***^p < 0.001).

**Figure 7:**
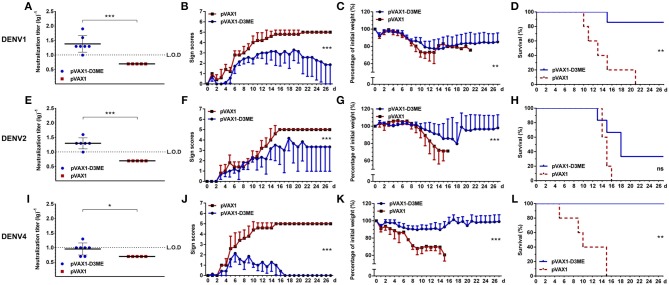
Long-term cross-reactive NAb response and protection against DENV1 **(A–D)**, DENV2 **(E–H)**, and DENV4 **(I–L)** at the 12th month after the immunization. For DENV1, *n* = 7 in the pVAX1-D3ME group, *n* = 5 in the pVAX1 group. For DENV2, *n* = 6 in the pVAX1-D3ME group, *n* = 5 in the pVAX1 group. For DENV4, *n* = 7 in the pVAX1-D3ME group, *n* = 5 in the pVAX1 group. **(A,E,I)** Endpoint titers of cross-reactive NAbs in sera were detected by PRNT_50_ and recorded as GMT ± SD. The L.O.D. depicted as a dotted line represents the limit of detection of the assay. **(B–D,F–H,J–L)** Mice were challenged with DENVs and monitored daily for 27 days. **(B,F,J)** Pathological symptoms recorded as the mean clinical sign scores. **(C,G,K)** Percentage of body weight from day 0. **(D,H,L)** Survival rate shown as the percentage of survivors. *p < 0.05; **p < 0.01; ***p < 0.001.

At the 12th month, the cross-reactive NAb titers decreased to 1:24.38, 1:20, and 1:9.06 (Figures 7A,E,I). After challenged with DENV1 and DENV4, the mice immunized with pVAX1-D3ME showed 85.7% (6/7, Figure 7D, ^**^p < 0.01) and 100% (7/7, Figure 7L, ^**^p < 0.01) survival rates, respectively. Meanwhile, the symptoms were relatively mild (Figures 7B,J, ^***^p < 0.001) and the levels of the body weight loss were less than those of the controls (Figure 7C, ^**^p < 0.01; Figure 7K, ^***^p < 0.001). After challenge with DENV2, when compared with that of the control, although the survival rate of the pVAX1-D3ME-immunized mice was 33.33% (2/6) without statistic difference (Figure 7H, *p* = 0.0564), differences in symptom scores and body weight loss (Figures 7F,G, ^***^p < 0.001) indicated the partial cross-reactive protection.

The aforementioned results demonstrated that different degrees of the cross-reactive protection evoked by three doses of pVAX1-D3ME could last up to 12 months from the initial immunization.

## Discussion

Dengue disease is the most common mosquito-borne viral disease in the world. Severe dengue is a leading cause of death among children in Southeast Asian and Latin American countries (Kittigul et al., 2007). In China, the region affected by dengue has expanded, and the incidence has increased steadily since 2012 (Lai et al., 2015). In 2014, Guangdong Province in China suffered the most serious dengue outbreak in its history, with more than 60,000 cases (Zhu et al., 2019). The development of virus-specific prevention is an urgent public health priority to control the increased global incidence and to reduce the disease burden. At present, only Dengvaxia, Sanofi's controversial dengue vaccine, has been developed for use in humans. Recently, Dengvaxia was approved for the prevention of secondary DENV infection in individuals 9 through 16 years of age who have had laboratory-confirmed dengue disease and who live in endemic regions (FDA, 2019). However, the recommendation for extra safety precautions for this vaccine by the WHO (Sridhar et al., 2018) points to the limitation of its application. Another live-attenuated tetravalent dengue vaccine, TAK-003, from Takeda in Japan was reported to have positive top-line results from a very large Phase III trial (Biswal et al., 2019; Sharma et al., 2019; Tong, 2019). Nevertheless, developing efficacious vaccines against dengue with balanced immune responses, especially tetravalent protection, is of great necessity.

As a novel and rapidly developing approach, DNA vaccines offer a number of potential advantages including cost-effectiveness, simplicity of manufacturing, high safety, and long-term expression of immunogens (Khan, 2013). More importantly, antigens encoded by DNA vaccines can be processed through either the MHC class I or II pathway, which are capable of stimulating robust cellular and humoral immune responses. Moreover, DNA vaccines have been reported to trigger balanced immune responses and provide effective protection against DENVs (Beaumier et al., 2013). Previously, we confirmed the effective protection induced by recombinant plasmids expressing the prM and E proteins of DENV1 (Zheng et al., 2017), DENV2 (Chen et al., 2016), or DENV4 (Sheng et al., 2019). With the same dosage and immunization strategy, two or three weeks after the third vaccination, the survival rates against lethal challenge with DENV1, 2, 3, and 4 were 100, 90, 100, and 100%, respectively, and the protective efficacy against all four serotypes was stable.

The effective protective immunity evoked by recombinant plasmids expressing the prM and E proteins of DENVs should first be attributed to the target molecules used in the vaccine. It has been demonstrated that prM and E proteins are involved in inducing effective NAbs to DENV (Lin et al., 2015). Additionally, T cell epitopes eliciting cellular immune responses may also be important. Although the vast majority of T cell epitopes have been identified on non-structural proteins, especially NS3 (Mathew et al., 2014; Elong Ngono et al., 2016), they have also been found on structural proteins, including prM and E (Duan et al., 2015; Hussain et al., 2015; Wen et al., 2017). Theoretically, DNA vaccine-encoded proteins often display a native conformation with post-translational modifications, including glycosylation, proteolytic processing, and lipid conjugations, which are essential for eliciting immune responses to conformational epitopes. Moreover, it has been demonstrated that electroporation delivery could facilitate DNA vaccination to generate a significantly persistent antibody response and antigen-specific T cell response (Chen et al., 2016; Zheng et al., 2017; Sheng et al., 2019), which would contribute to the effective protection induced by pVAX1-D3ME.

Although the amino acid sequence of the E protein defines each DENV serotype, the amino acid residues of this protein are well-conserved, with high similarities (90%–96%) among the four different serotypes (Cedillo-Barron et al., 2014). Moreover, it has been reported that anti-prM antibodies exhibit distinctive cross-reactivity to the four DENV serotypes (Chan et al., 2012). In this study, pVAX1-D3ME-elicited antibodies showed cross-neutralizing and durable reactivity toward heterologous DENVs. More importantly, most of the pVAX1-D3ME-vaccinated mice survived the challenge with heterologous serotypes, without an increase in viral infection. Speculatively, both of the cross-reactive NAbs and CD8^+^ T cells might contribute to the cross-protection against heterotypic DENV infection (Zellweger et al., 2015). Our results are consistent with a previous study from Reich et al., who reported that infection or immunization with one DENV serotype conferred effective cross-protection to heterologous serotypes for an average duration of approximately two years (Reich et al., 2013). Thus, the characteristics of cross-reactive immunity to DENVs and its impact on dengue epidemics need to be further investigated.

Several vaccines specifically targeting DENV3 have been developed and evaluated in murine models. Most of them expressed E protein as the specific immunogen. Chiang et al. designed a subunit vaccine expressing recombinant DENV3 E protein domain III in a lipidated form (LD3ED III) and confirmed that LD3ED III induced broad profiles of humoral and cellular immune responses even without formulation with exogenous adjuvants (Chiang et al., 2016). Versiani et al. used a diimide-activated amidation process to bind recombinant DENV3 E proteins and developed multiwalled carbon nanotubes. The generation of both cell-mediated and NAb responses against DENV3 increased substantially (Versiani et al., 2017). Hurtado-Melgoza et al. reported that the DNA vaccine candidate targeting DENV3 NS3 triggered a favorable response with the activation of T lymphocytes; however, immunization with this DNA vaccine presented no detectable antibody titers against DENV3 (Hurtado-Melgoza et al., 2016).

Regarding the limitations of this study, the possibility of increasing immunogenicity with a lower dose of DNA than 50 μg of each immunogen and a different regimen should be considered. In our previous work, based on a consensus sequence of the ectodomain of E protein (cE80) of the four DENV serotypes (Wang et al., 2019), heterologous (DNA prime-protein boost) regimens elicited greater systemic immune response and more effective tetravalent protection than did homologous DNA immunization. As the optimal immunization regimen, DNA vaccination followed by protein boosting has been applied to a variety of infectious agents to improve the immunogenicity and protective efficacy of vaccines (Menon et al., 2017; Wang et al., 2017; Cai et al., 2018). Further investigation should be performed to assess whether it is an appropriate strategy for our dengue DNA vaccine candidate.

Taken together, this study demonstrated that three doses of 50 μg of pVAX1-D3ME were sufficient to induce robust NAbs and strong antigen-specific T cell responses and provided long-term protective immunity against DENV3 infection. Moreover, pVAX1-D3ME-elicited NAbs and protective immunity were not only type specific but also cross-reactive against heterotypes, showing great promise for the further evaluation of a dengue tetravalent DNA vaccine candidate. In a future study, immunogenic motifs in the DENV proteins that are conserved or highly homologous among all serotypes should be considered alternative immunogens in a dengue tetravalent vaccine formulation.

## Data Availability Statement

The raw data supporting the conclusions of this article will be made available by the authors, without undue reservation, to any qualified researcher.

## Ethics Statement

This animal study was reviewed and approved by Institutional Animal Care and Use Committee of Chinese Capital Medical University.

## Author Contributions

JA and HC conceived and designed the experiments. KF, XZ, RW, DF, and ZS performed the experiments. KF, NG, HC, and JA analyzed the data. HC and KF prepared the manuscript draft. HC, JA, and HZ revised the manuscript. All authors read and approved the final manuscript.

### Conflict of Interest

The authors declare that the research was conducted in the absence of any commercial or financial relationships that could be construed as a potential conflict of interest.
